# Development of 3D human intestinal equivalents for substance testing in microliter-scale on a multi-organ-chip

**DOI:** 10.1186/1753-6561-7-S6-P65

**Published:** 2013-12-04

**Authors:** Annika Jaenicke, Dominique Tordy, Florian Groeber, Jan Hansmann, Sarah Nietzer, Carolin Tripp, Heike Walles, Roland Lauster, Uwe Marx

**Affiliations:** 1 TU Berlin, Institute for Biotechnology, Faculty of Process Science and Engineering, 13355 Berlin, Germany; 2 TissUse GmbH, 15528 Spreenhagen, Germany; 3 Fraunhofer Institute for Interfacial Engineering and Biotechnology IGB, 70569 Stuttgart, Germany; 4 Chair of Tissue Engineering and Regenerative Medicine, Julius-Maximilians-Universität Würzburg, 97070 Würzburg, Germany

## Background

Robust and reliable dynamic bioreactors for long term maintenance of various tissues at milliliter-scale on the basis of a biological, vascularized matrix (BioVaSc^®^) have been developed at the Fraunhofer IGB in Stuttgart, Germany. As an intestinal in vitro equivalent, seeding of the matrix with CaCo-2 cells yielded in the self-assembly of a microenvironment with the typical histological appearance of villus-like structure and morphology [1]. We modified this matrix (BioVaSc^®^) - cell (CaCo-2) system to some extent with the aim to develop 3D intestinal equivalents for systemic preclinical testing of orally applied drug candidates in microliter-scale on a human Multi-Organ-Chip (MOC), which consists of different organ equivalents important for ADMET (adsorption, distribution, metabolism, excretion, toxicity) testing.

## Materials and methods

**Figure 1:**
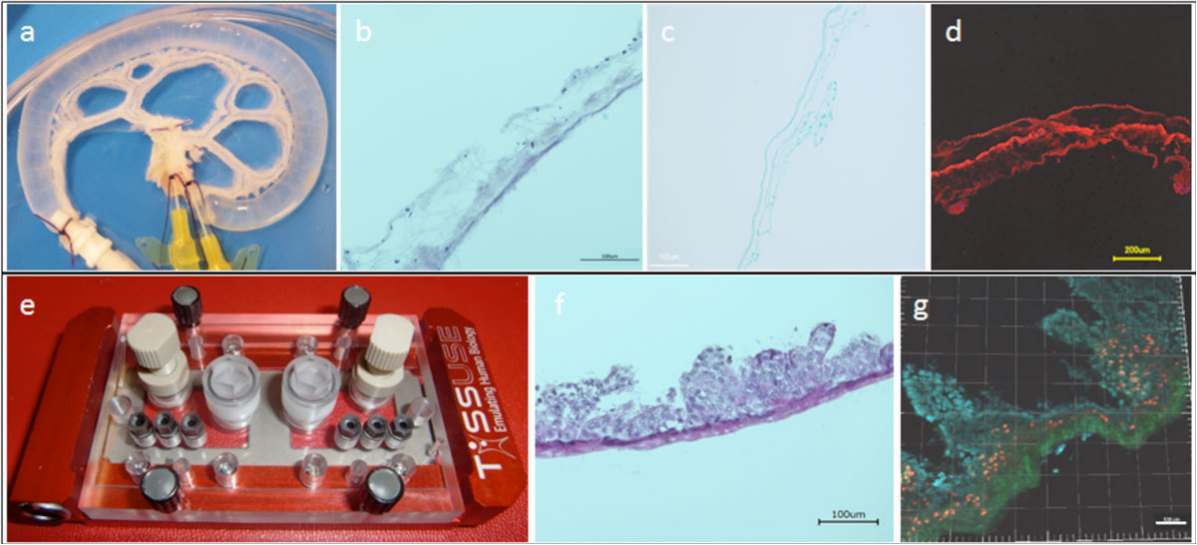
**a-d) Characterization of the decellularization procedure**. **a) **Explanted jejunal segment with the preserved capillary bed after decellularization. **b) **H/E staining of the decellularized matrix. **c) **Feulgen staining of the decellularized matrix. **d) **immunofluorescent stainings for collagen I on rBioVaSc. **e) **The multi-organ-chip (MOC) device consisting of an integrated micro-pump, a microfluidic .channel system and inserts for the cultivation of different organ equivalents. **f+g) **Characterization of the intestinal in vitro equivalent. **f) **H/E staining of the recellularized matrix after one week of dynamic culture in the MOC device. **g) **Second Harmonic Generation by 2 PM, nuceli were stained with Hoechst 33342.

For the generation of biological, vascularized matrices (rBioVaSc^®^), jejunal segments of the small intestine of Wistar rats including the corresponding capillary bed were explanted and decellularized by perfusion with 1% sodium deoxycholate. Characterization of the matrix was done by histological analysis as well as 2-photon microscopy (2 PM) and immunofluorescent stainings. After sterilization by γ-irradiation, the rBioVaSc^® ^could be used to built up a 3D intestinal equivalent. Punch biopsies of the matrix were fixed on the frame of a 96-well transwell insert and seeded with CaCo-2 cells (2*10^6 cells) on the former luminal side of the matrix following static cultivation for 48 hours and integration in a perfused MOC device. Our MOC device consists of an integrated micro-pump, a microfluidic channel system and inserts for the cultivation of different organ equivalents (Figure 1e). For the generation of the intestinal equivalent, the generated matrix-cell construct was placed in the MOC device and perfused for up to one week with cell culture medium (supplemented MEM), following histological as well as immunofluorescence (IF) analysis of the growth behavior of the cells. As a control, matrix-cell constructs were cultivated statically. Daily medium samples have been analyzed to monitor metabolic activity and the absorption properties of the intestinal equivalent. Immunohistostaining of cryo-preserved tissue slices have been analyzed to compare self-assembled organoid tissue structures with their corresponding in vivo counterparts.

## Results

Decellularization of jejunal segments of rats together with the corresponding capillary bed yielded in a biological, vascularized matrix which was free of non-human cells but with the preserved 3D structure of the former intestinal extracellular matrix (ECM) (Figure 1a-d). Those ECM components were used for the resettlement of human intestinal cells (CaCo-2) which resulted in the formation of characteristical villus-like structures on the matrix after one week of perfused cultivation (Figure 1f+g). Cells expressed typical intestinal epithelial markers, e.g. CK8/18, EpCAM and Na/K-ATPase. Process parameters, such as nutrient perfusion rate and culture time, have been optimized to qualify the system for repeated dose testing of orally administered drug candidates.

## Conclusions

As shown by histological as well as immunofluorescent stainings, we succeeded in the development of self-assembled 3D organ equivalents which have a characteristical intestinal architecture. Those organ equivalents can be used as an in vitro system for the evaluation of adsorption properties of orally administered drugs in microliter-scale on a multi-organ-chip (MOC). Further improvements of the MOC device are necessary, e.g. the integration of a second circulation, representing the intestinal lumen. In addition, reseeding the matrix with primary intestinal cells as well as co-cultures of epithelial and endothelial cells are planned.

## References

[B1] Pusch J Votteler M Göhler S Engl J Hampel M Walles H Schenke-Layland K The physiological performance of a three-dimensional model that mimics the microenvironment of the small intestine Biomaterials 2011 7 7469 7478 21764120 10.1016/j.biomaterials.2011.06.035

